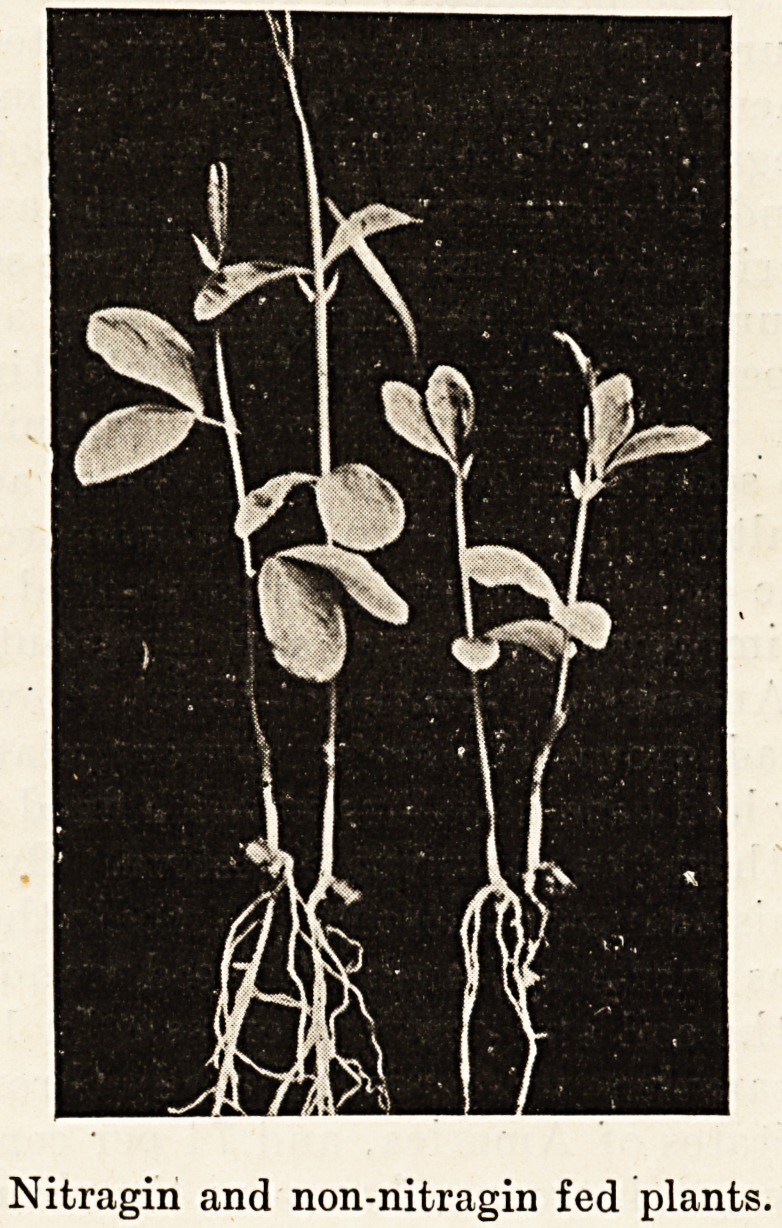# Nature's Method and the Food Supply

**Published:** 1907-10-26

**Authors:** 


### NATURE'S METHOD AND THE FOOD SUPPLY.

As long ago as 1898 Sir William Crookes estimated that within the next quarter of a century there would be an increase of 230 million mouths to feed with cereals. This means that 330 million more bushels of wheat will be required to feed them. Since increase of the population means decrease of arable land, starvation seems to be staring the world in the face. This crisis, it appears, can be averted by the devotees of science. The realisation of this desired result has been made possible by the production from air, water, and lime of nitrate of lime or Norwegian saltpetre in the factory of Birkeland and Eyde, at Christiania, where the electric arc in the magnetic field is utilised.

More recently we have the statement made by two [illegible]ofessors of the Catholic University of Fribourg, in Switzerland—the town of the famous linden tree —that they have succeeded in manufacturing nitric acid from atmospheric nitrogen. This is of great national importance, since there is at present only a definite amount of combined nitrogen available for the food supply of the world. By the ordinary metabolism of the human body and by decomposition, this is gradually being reduced to its free state, a condition in which it is not available as food. For purposes of growth animals require nitrogen in the shape of proteids. Plants, on the other hand, utilise nitrates, and out of this they manufacture the proteids necessary for animal life. Hence any method which will produce nitrates in a simple and economical manner has an important bearing on the food supply of the world, especially in those most important parts of the world where a meat diet is

Plant (left-hand figure) grown under ordinary conditions, contrasted with one (right-hand figure) fertilised with nitragin.

considered essential. Where work requires to be done a plentiful supply of proteids is needed.

Cavendish showed first of all that the free nitrogen of the atmosphere might be made to combine with oxygen to produce nitrous fumes by the passage of an electric spark through an ordinary atmosphere. Nature has discovered a method of her own by which free nitrogen can be restored to a com-

Nitragin and non-nitragin fed plants.

bined state available alike for animal and plant food; this is done by certain organisms known as nitrogen fixing organisms. These beneficent organisms bring the free nitrogen back into Nature's cycle.

The fact that the growing of leguminous crops added a large amount of nitrogenous material to the soil was well known to the ancients. Pliny wrote: "The bean ranks first among the legumes, and fertilises the ground in which it has been sown better than any manure." Ancient writings make frequent reference to the necessity of including some leguminous crops in the ordinary rotation. The exact meaning of this was not known until recent times, when it was shown by Hellriegel in 1886 that the atmosphere was the source of nitrogen for leguminous plants. Sea plants possess nodules on their roots, described by the famous physiologist Malpighi in 1687 as gall-formations, but it was not until 1866 that these were found to contain living organisms, which were accurately described by Woronin. The growth of leguminous plants in soil free from nitrogen always depends upon the development of similar nodules on the root. This was demonstrated by Willfarth, and in the same year Beyerinck was able to grow pure cultures of the bacteria. This micro-organism was first described as a bacillus; it is now named psue radicicola. It has three well-defined forms:—(1) A minute motile rod which is active in penetrating the root hair of the plant; (2) a rod form varying from .6 $\mu$ to 2.5 $\mu$ in width and 1.5 $\mu$ to 5.0 $\mu$ in length; (3) branched forms Y- and V-shaped to which the name of bacteroids is given.

Professor Nobbé, experimenting with pure cultures, demonstrated the value of these bacteria for leguminous crops, and as the result of a series of experiments he prepared a pure gelatine culture of bacteria from root nodules to which he gave the name of "*Nitragin.*"

This was tried at several agricultural colleges and declared to be practically a failure. In 1900 the Agricultural Department of the United States Government sent over an expert to confer with Nobbé regarding the possible success of soil inocculation, and the former did not report favourably. The American Government, however, was so assured of the soundness of the theory that they appointed four experts to devote their whole time to a thorough investigation of these organisms and to evolve some method that might be of advantage to agriculture. After two years' research and a multitude of experiments they succeeded in working out improved cultures, and distributed them among American farmers. They showed that Nobbé had grown the organisms on gelatine, and that they had become saturated with fixed nitrogen, and had therefore ceased to be active. By growing the organisms in non-nitrogenous media their fixing power was increased, and produced wonderful results on the soil and growing crops. In 1903 over 12,000 samples were distributed through the various States of America, and 74 per cent. of the trials gave an increase of crop as the result of the inocculation. In England, culture material obtained from the American Government has been distributed by the Board of Agriculture to 13 different agricultural colleges and experimenting stations. The results were in most cases unsatisfactory, and it fell to Professor Bottomley, of King's College, to demonstrate that the treatment to which the cultures had been subjected in this country had rendered the bacilli either dead or comatose, or at all events inactive. Professor Bottomley was engaged in working out certain points in the life-history of this organism, and, being convinced by experiments of the value of soil inoculation, he took care in distributing his samples that they should be used fresh, and in almost every instance where results were obtained the advantage of inoculation was evident, and farmers were quick to recognise it. The applications received at King's College Laboratory are far more numerous than can be dealt with.

The method of application is simplicity itself. Three packages are provided: The first contains maltose, potassium phosphate, and magnesium sulphate—the nutrient medium in which the bacteria grow; the second contains the bacteria; the third contains ammonium phosphate to stimulate the growth. Packet No. 1 is dissolved in a gallon of water, then No. 2 is put in it, and at the end of 24 hours No. 3 is added as a tonic. At the end of the second 24 hours a milky-white fluid is produced —the outward and visible sign of the inward multiplication of the bacteria. With this quantity two acres of land can be inoculated. The seed can be inoculated if it is placed in the culture solution and dried in a shady place. The results are astonishing. Thus three-quarters of a pound of inoculated seed give more peas than one and a half pounds of uninoculated. Beans grown in a soil that had not been manured for ten years increased 43 per cent., and peas that were inoculated were ready for the market three weeks sooner, and were 50 per cent. more prolific.

One important advantage of the bacterial treatment of soil over that by nitrate of soda, is that the latter is absorbed by the crops, and the land becomes exhausted of its nitrogen, whereas the former doubles the crop, and further, owing to the nodules which remain behind in the soil, increases the nitrogenous contents by 200 pounds of nitrogen per acre; the equivalent, that is to say, of half a ton of nitrate of soda is available for the succeeding crop.

The results of recent experiments indicate that by this method of inoculation not only is the yield of the leguminous plants increased, but nitrogen is stored up in the soil for the use of succeeding crops. More important than that, the food value of the produce is greatly increased. Thus experiment has shown that tares manured with nitrate of soda (2 cwt. to the acre) yielded 1.92 per cent. of nitrogen, but when treated by the inoculation method, the yield of nitrogen was 3.07 per cent., that is, 50 per cent. more than in the case of those grown with the assistance of the nitrate of soda. The value of these foods naturally depends on the proteid content of the grain.

## Figures and Tables

**Figure f1:**
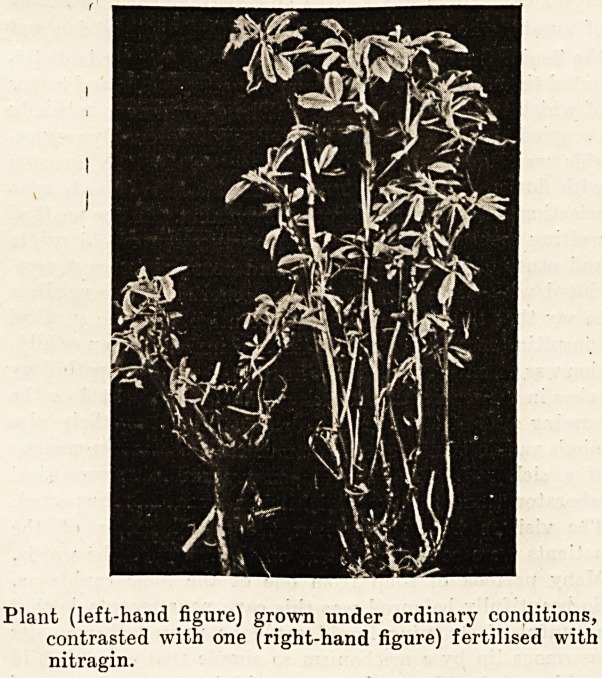


**Figure f2:**